# An Urticarial and Acral Manifestation of Bullous Pemphigoid After Vildagliptin Therapy: A Case Report

**DOI:** 10.7759/cureus.57054

**Published:** 2024-03-27

**Authors:** Khushboo Bisht, Surajbala Khuraijam, Hannah Theresa Sony, Saurabh Kumbhar

**Affiliations:** 1 Clinical Pharmacology, All India Institute of Medical Sciences, Rishikesh, Rishikesh, IND; 2 Dermatology, All India Institute of Medical Sciences, Rishikesh, Rishikesh, IND; 3 Pharmacology, All India Institute of Medical Sciences, Rishikesh, Rishikesh, IND; 4 Internal Medicine, All India Institute of Medical Sciences, Rishikesh, Rishikesh, IND

**Keywords:** dpp-4 inhibitors, drug-induced bullous pemphigoid, elderly, vildagliptin, bullous pemphigoid

## Abstract

Drug-induced bullous pemphigoid (DBP) may present identical to an idiopathic type of illness. Thus, DBP should be considered as a differential diagnosis when evaluating the cause of bullous pemphigoid (BP) in elderly individuals with diabetes. We present the case of a 65-year-old "young-elderly" female who developed bullous lesions after initiation of medication. This case report casts light on a commonly prescribed antidiabetic drug, a dipeptidyl peptidase-4 inhibitor, vildagliptin, and its unforeseen complication in the elderly. The long-term complication is BP. With long-term use, there is an increased likelihood of encountering such cases among the elderly. Thus, we recommend that DBP be considered an important early differential diagnosis among elderly diabetics presenting with initial signs and symptoms of BP.

## Introduction

Bullous pemphigoid (BP) is an autoimmune subepidermal blistering skin condition caused by antibodies against the hemidesmosome proteins BP180 and BP230 [1]. Blisters frequently precede irritating urticarial or eczema-like lesions, which are typical for BP [2]. Usually, a prodromal phase is followed by the eruption of tense blisters. This prodromal phase may present with eczematous or urticarial lesions. Due to the availability of precise laboratory techniques to show the presence of autoantibodies against hemidesmosome proteins, diagnosis of BP can be easily confirmed. The estimated yearly incidence in Asia is between 2.6 and 7.5 cases per million [3]. Drugs, thermal or electrical burns, surgeries, trauma, ultraviolet radiation, radiotherapy, chemical preparations, transplants, and infections are some of the triggers that can cause or worsen BP disease [4].

The existing knowledge regarding the pathophysiology of drug-induced BP is limited [5]. Drug-associated BP (DABP) refers to cases of BP, which have clinical, histological, or immunopathological characteristics that are identical to or comparable to those of the idiopathic type of the illness. DABP may be related to topical or systemic use of specific medications [6]. Dipeptidyl peptidase-IV (DPP-4 or CD26) inhibitors, which are commonly used anti-diabetes medications (DM), are now recognized as a potential risk factor for causing BP [2].

DPP-4 inhibitors are presently recommended as second-line pharmacological therapy for people with type 2 diabetes, along with metformin [7]. The median interval between the start of BP and the delivery of DPP-4 inhibitors was about nine months, ranging between two and 13 months [8]. We present one such case of vildagliptin-induced BP. Interestingly, the management of BP was different from previously reported cases.

## Case presentation

A 65-year-old patient has been suffering from type 2 diabetes since 1992. This patient also had a history of dyslipidemia. The patient was prescribed a new drug vildagliptin 50 milligrams every 12 hours, while the patient was concurrently on 10 milligrams of dapagliflozin every 24 hours before breakfast, a subcutaneous injection of Lantus 30 units at bedtime, and subcutaneous regular insulin before breakfast, lunch, and dinner. This patient had a history of hypertensive disorder and was well-controlled on amlodipine 5 mg for five years. There was no history of any microvascular or macrovascular pathology.

The patient visited the dermatology outpatient department with complaints of itching and rashes on and off for a month. The physician prescribed emollient and antihistaminic following which all complaints were relieved. Thus, the initial presentation was non-bullous. One day, the patient visited the dermatology outpatient department with a sudden eruption of development of diffuse, tense, bullous, itchy lesions over the entire body. On that same day, the patient was admitted to the dermatology ward for a detailed examination and workup.

**Figure 1:**
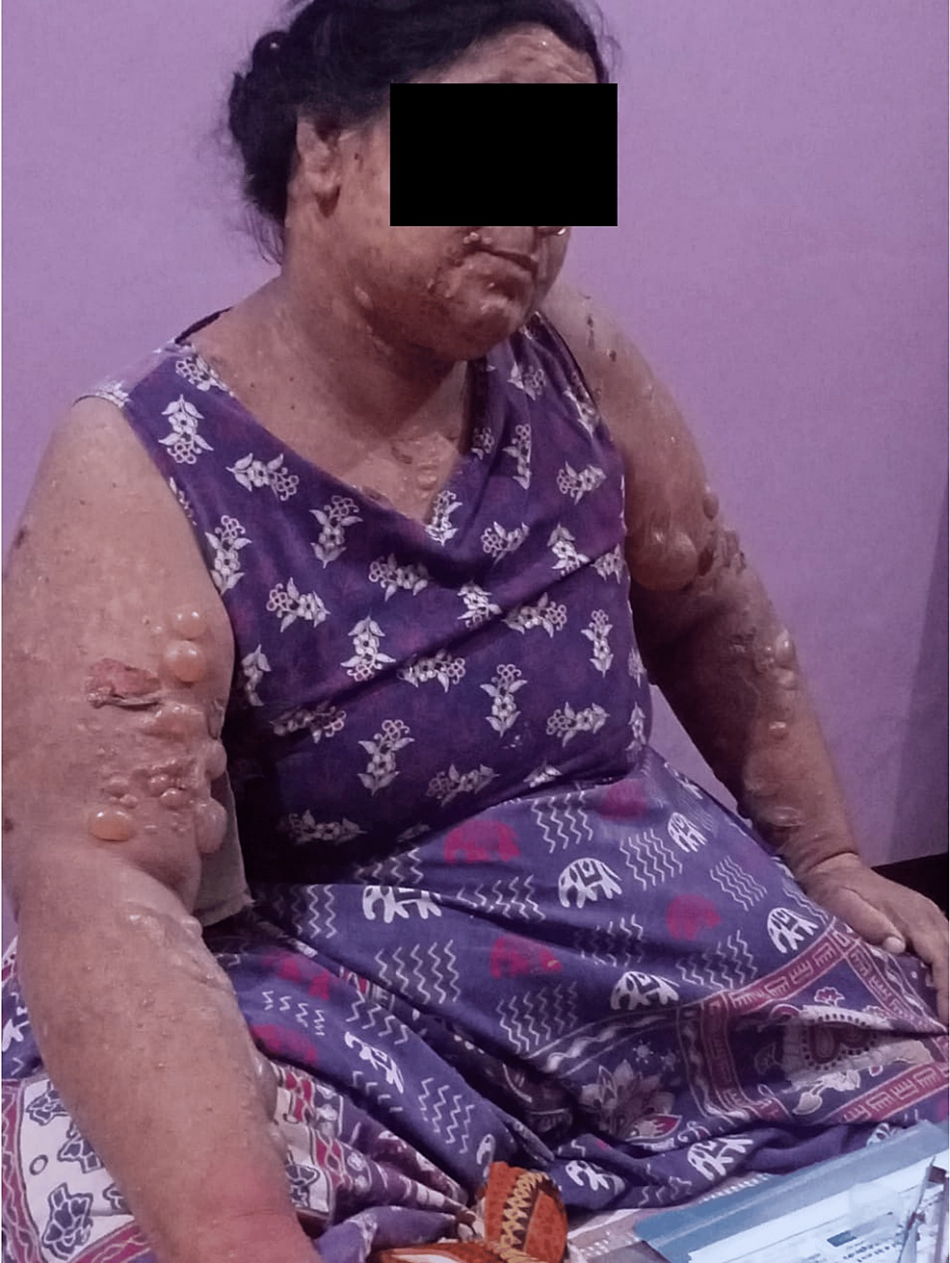
Cutaneous eruptions of tense, erythematous, and non-erythematous bullae

On physical examination, vitals were found to be stable with no other systemic abnormalities. Cutaneous examination revealed multiple tense bullae of size ranging from 1 x 2 cm to 4 x 3 cm over non-erythematous and erythematous bases present predominantly on the upper extremities, trunk, face, and lower limbs (Figure 1). Few erosions and urticarial plaques were also seen. Thus, a mixed presentation of urticarial followed by an acral form of BP was observed. Also, vesicular forms of BP, which are less common, were seen over the lips and oral cavity of this patient.

Diagnosis

Nikolsky sign was positive. Skin biopsy showed subepidermal vesicles with dyskeratotic keratinocytes and inflammatory infiltrate with marked eosinophils, neutrophils, and lymphocytes. Direct immunofluorescence (DIF) shows linear IgG and C3 deposition along the dermo-epidermal junction as in BP. Tzanck smear was negative for giant cells and acantholytic cells.

Random blood sugar was stable at 220 mg/dL. A bedside urinalysis revealed 3+ glucose levels. All viral markers were negative. All routine investigations were within acceptable ranges, but the total blood count revealed eosinophilia. HbA1c was 8.6%.

Initially, we did not suspect the urticarial form of BP; however, upon serious manifestation, a diagnosis of bullies pemphigoid was established. Based on the skin biopsy report and DIF findings, the diagnosis of drug-associated BP (DABP) was confirmed.

Case management

**Figure 2:**
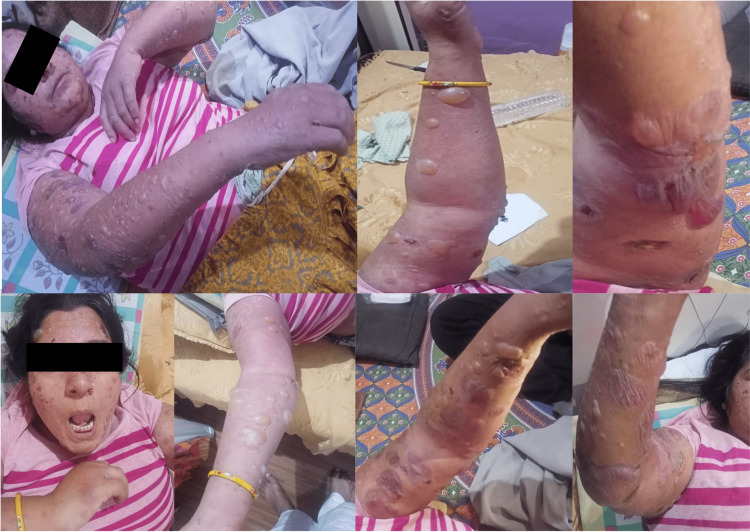
Slowly resolving lesions of acral bullous pemphigoid

Vildagliptin drug was discontinued, and oral prednisolone was started at a dose of 1 mg/kg/day with topical steroids for local application on lesions. Concomitantly, the patient received 500 mg of tetracycline twice daily for seven months and 75 mg of azathioprine twice daily for one month followed by azathioprine 50 mg twice daily for the next six months. Also, the patient received a single dose of 40 mg intravenous immunoglobulin along with azathioprine. Lantus and regular insulin were continued during the first month after BP, and the lesions started resolving (Figure 2).

Follow-up and outcome

**Figure 3:**
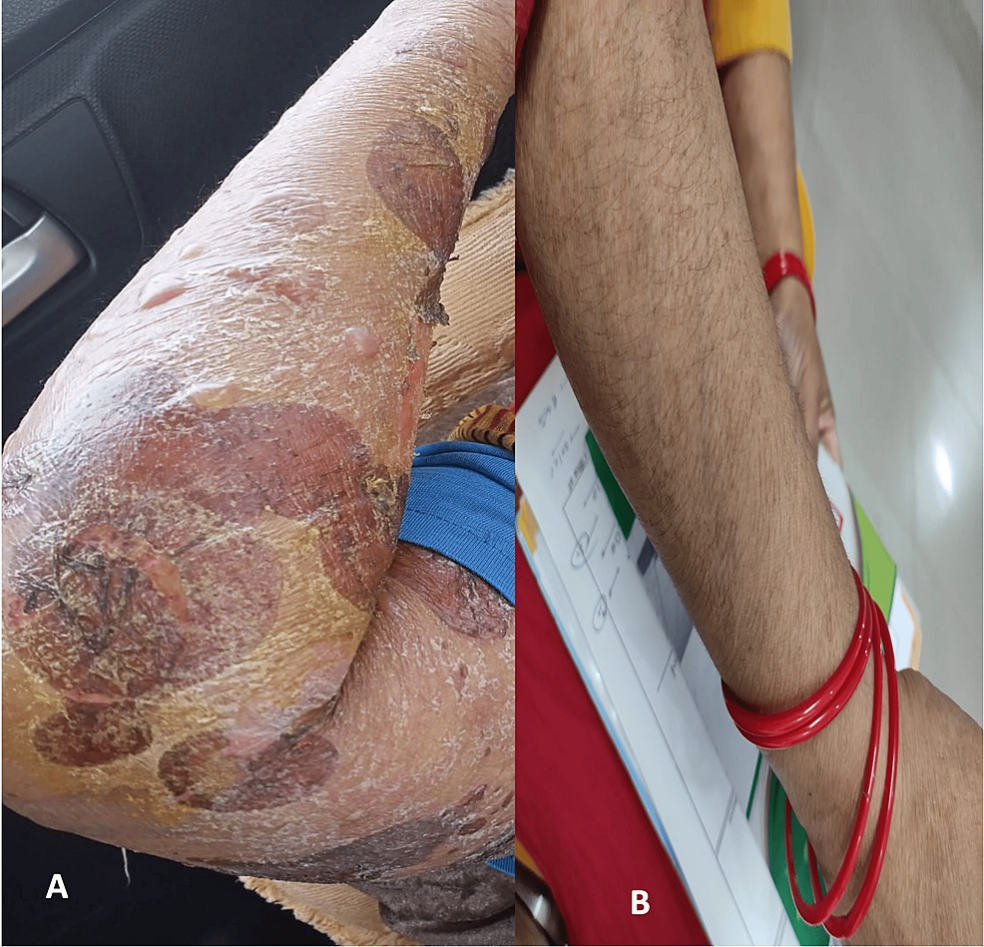
From left to right (A and B), resolved lesions can be identified, with part A showing healing lesions of bullous pemphigoid at one month and part B showing complete healing of lesions after seven months of treatment

On follow-up after one month, the patient dramatically improved with the BP lesions, and no new lesions appeared (Figure 3, Panel A). Vildagliptin discontinuation along with steroids led to a partial control of BP at one month. On follow-up visits, the steroid was tapered, and all the lesions were healed in seven months (Figure 3, Panel B).

## Discussion

DPP-4 inhibitors have gained popularity as a second-line type 2 diabetes treatment because they do not cause weight gain and have fewer side effects than sulfonylureas. BP and DPP-4, however, have been linked to an increasing number of case reports and epidemiological studies published during the past 10 years [9]. DPP-4 inhibitors have been linked to immunological conditions such as nasopharyngitis, inflammatory bowel disease, pancreatitis, hepatitis, and myalgia as well as hypersensitive skin reactions like anaphylaxis and angioedema [10].

BP is mainly seen in elderly patients with a mean age of onset between 69 and 83 years [11]. In this case, the younger age of the patient and the rapid response on discontinuation of the drug with no relapses on subsequent visits strongly indicate DABP. However, at the initial presentation, we could not suspect BP as the presentation was of urticaria. Thus, elderly diabetics on DDP-4 inhibitors presenting with urticaria should be suspected and followed for the development of BP.

Other features supporting this case were skin biopsy and DIF findings. More research involving clinical surveillance and studies is required to fully comprehend the mixed presentation of DABP as an acral or urticarial form of BP.

Although it is already known that DPP-4 inhibitors can cause BP, a mixed presentation is unlikely. Also, missing an early diagnosis of DABP incurs a heavy cost to the patient and the hospital.

A mean duration of eight months is needed for remission when treated with corticosteroids and doxycycline [12]. Skandalis et al. were the first to note that patients who were exposed to DPP-4 inhibitors had a risk of developing BP [10]. Case-control research comparing 328 age- and sex-matched controls with diabetes who had no BP to 82 diabetic patients who had BP in 2018 found a greater correlation between DPP-4 inhibitors and BP. Sitagliptin showed a weaker correlation than vildagliptin and linagliptin. Vildagliptin was a suspected drug in this case only when the patient presented with severe manifestation. However, fortunately in our case as compared to the usual treatment, stopping DPP-4 inhibitors had a superior result [13]. This case report adds to the evidence in favor of BP as a detrimental effect of vildagliptin and alerts physicians to consider when elderly diabetics visit with similar mixed presentation of BP [5].

## Conclusions

In conclusion, this case report underscores the importance of recognizing drug-induced BP as a potential complication in elderly diabetic patients treated with DPP-4 inhibitors. Heightened clinical suspicion and prompt diagnosis are crucial for effective management and prevention of severe outcomes. Interdisciplinary collaboration is essential for timely intervention and optimal patient care. Further research is needed to investigate the pathophysiological mechanisms and risk factors associated with DABP development. By implementing proactive measures, clinicians can improve outcomes and enhance the quality of care for affected individuals.
